# An eHealth program versus a standard care supervised health program and associated health outcomes in individuals with mobility disability: study protocol for a randomized controlled trial

**DOI:** 10.1186/s13063-018-2646-z

**Published:** 2018-04-27

**Authors:** Daniel Berglind, Gisela Nyberg, Mikaela Willmer, Margareta Persson, Michael Wells, Yvonne Forsell

**Affiliations:** 1 Department of Public Health Sciences, Karolinska Institutet, Stockholm, Sweden; 2 Department of Health and Caring Sciences, University of Gävle, Gävle, Sweden; 3 Department of Nursing, Umeå Universiy, Umeå, Sweden

**Keywords:** Mobility disability, Physical activity, Randomized controlled trial (RCT), Exercise, Fitness

## Abstract

**Background:**

Young adults with mobility disability (MD) are less likely to engage in regular physical activity (PA) compared with their able-bodied peers and inactive adults with a MD are more likely to report one or more chronic diseases compared to those who are physically active. Despite the vast amount of research published in the field of PA interventions over the past decades, little attention has been focused on interventions aiming to increase PA among individuals with MD. Thus, we propose to compare the effects of an eHealth program compared to a usual care supervised health program on levels of PA and other health behaviors.

**Methods:**

The current intervention will use a randomized controlled trial (RCT) design with two treatment groups (an eHealth program and a usual care supervised health program) in young adults with newly acquired MD. In total, 110 young adults (aged 18–40 years) with a MD, acquired within the past 3 years, will be recruited to participate in a 12-week intervention. The primary study outcome is accelerometer-measured time spent in moderate to vigorous PA. Secondary outcomes includes health-related quality of life, depression, stress, fitness, body composition, diet, musculoskeletal pain, motivation to exercise and work ability.

**Discussion:**

There is a lack of RCTs investigating effective ways to increase levels of PA in young adults with MD. Increased levels of PA among this physically inactive population have the potential to substantially improve health-related outcomes, possibly more so than in the general population. The trial will put strong emphasis on optimizing exercise adherence and investigating feasibility in the two treatment programs. The Ethical Review Board (EPN) at Karolinska Institutet has approved the study (2017/1206–31/1).

**Trial registration:**

International Standard Randomised Controlled Trial Number (ISRCTN), reference number ISRCTN22387524. Prospectively registered February 4, 2018

**Electronic supplementary material:**

The online version of this article (10.1186/s13063-018-2646-z) contains supplementary material, which is available to authorized users.

## Background

Approximately 10% of the Swedish population lives with a mobility disability (MD). MD is strongly associated with reduced work ability [1] and thus is a major public health concern. There are strong cross-sectional [2] and longitudinal [3] dose-response associations between physical activity (PA) and work ability, as well as health-related quality of life (HRQoL) [4]. Despite the numerous known health benefits of PA [5], young adults with MD are less likely to engage in regular PA compared with their able-bodied peers [6]. Approximately 50% of adults with MD are physically inactive, reporting little or no time spent in moderate to vigorous PA (MVPA) per day, compared to 26% of adults without MD [7]. In addition, inactive adults with a MD are 50% more likely to report one or more chronic diseases compared to those who are physically active [7].

Despite the vast amount of research published in the field of PA interventions over the past decades, little attention has been on interventions aiming to increase PA among individuals with MD [8]. Interventions focusing on PA for those with MD may be particularly important, as there is some evidence that a physically active lifestyle can provide increased health benefits for people with MD compared with the general population [9]. However, currently, PA interventions targeting individuals with MD are limited to older people [10]; therefore, there is a paucity of intervention research regarding the health benefits of PA for those with MD, especially for those who are of working age.

The few existing studies on PA and MD suggest that motivation for PA is high among individuals with MD, and that barriers to PA engagement include accessibility to tailored PA facilities, and they also have a lack of knowledge on how to engage in PA [6, 11]. In addition, enjoyment appears to be a critical individual factor for engagement in PA among these individuals [12]. A longitudinal study investigating motivation for PA, using self-determination theory, in young adults with MD indicates that autonomy, goal setting, surveillance, support and feedback are important factors for improving and maintaining healthy levels of PA [11]. Therefore, factors beyond health benefits should also be considered for helping those with a MD achieve greater levels of PA.

A recent systematic review found modest evidence for the efficacy of application interventions, that is app-based interventions, to improve the levels of PA and dietary habits for non-communicable disease prevention [13]. Given that many people have busy lifestyles, but still value access to health-behavior programs that provide advice, information, feedback and self-monitoring around the clock, app-based programs may be an important way to reach certain populations [14]. However, there is an inconsistency in the academic literature on the effects from multi-component versus eHealth app interventions on health outcomes [13]. This raises the question as to whether multi-component interventions deliver equal intervention effects compared with stand-alone app interventions.

Thus, the aim of the current randomized controlled trial (RCT) is to compare the effects of delivering an eHealth program compared to delivering a supervised health program on levels of MVPA, as well as evaluate the health-related benefits and cost-effectiveness to each program.

## Methods

### Intervention design

**Fig. 1:**
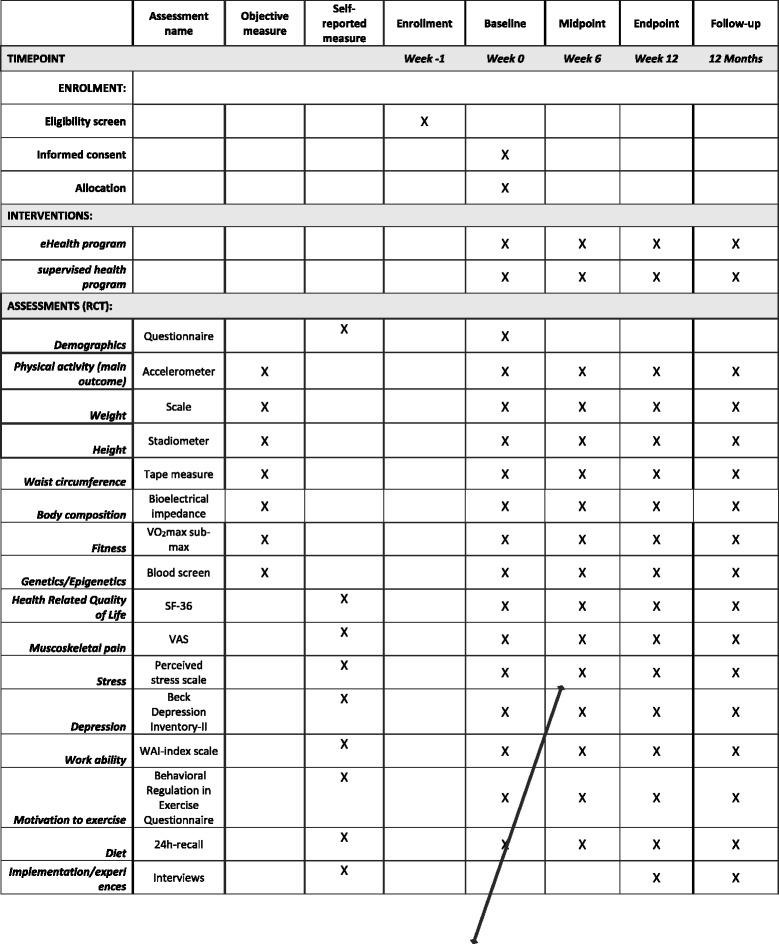
Schematic overview of the eHealth and the supervised health intervention groups

**Fig. 2:**
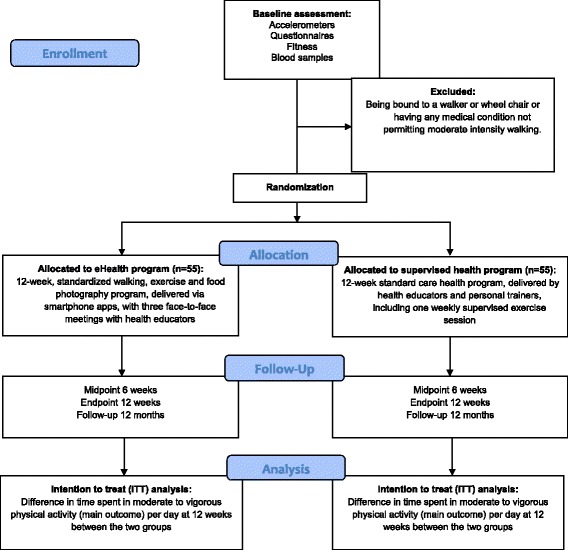
Participant flow diagram (according to Consolidated Standards of Reporting Trials (CONSORT) guidelines)

The current intervention will use a RCT design with two treatment groups in a sample of young adults with newly acquired MD. In this study, newly acquired MD refers to individuals who have had MD for 3 or less years. The trial will examine the efficacy of an eHealth program compared to a supervised health program, lasting for 12 weeks, on the primary (MVPA) and secondary (HRQoL, work ability, symptoms of depression, pain, stress, exercise motivation, diet, fitness, body composition and genetic/epigenetic factors) outcomes at baseline (week 0), midpoint (week 6), at the end of the intervention (week 12) and at 12 months’ post intervention follow-up to examine maintenance effects. In addition, semi-structured interviews focusing on facilitating and hindering factors associated with intervention adherence will be conducted at week 12 and at 12 months’ follow-up in a subsample of individuals from both intervention groups (illustrated in Fig. 1). All measurements, tests and information meetings will be held at a healthcare center (TWITCH Health Capital, Stockholm, Sweden). The trial will be reported according to Consolidated Standard of Reporting Trials (CONSORT) guidelines for reporting RCT designs [15] (Fig. 2). For Standard Protocol Items: Recommendations for Interventional Trials (SPIRIT) 2013 Checklist, see Additional file 1.

The two treatment arms are each designed to support participants to perform sustained changes in MVPA and health-related outcomes. The framework used in both intervention groups is based on the assumption that behavior-change interventions are more likely to be effective if they use intrinsic motivation strategies and are deep-rooted in health-behavior-change theory [16]. In addition, a recent meta-analysis supports the use of behavior-change techniques, including goal setting, self-monitoring and strategies for intrinsic motivation change of behavior, to promote change in healthy PA and dietary behaviors and a person-centered and autonomy supportive counseling approach in order to maintain these behaviors over time [17].

### The eHealth program

#### Short description

The eHealth program is a 12-week, standardized walking, exercise and food photography program, delivered via smartphone apps, with three face-to-face meetings with health educators (*n* = 55).

### Design

The intervention framework is based on a recent systematic review concluding that multi-component application-based interventions to improve diet, PA and sedentary behaviors can be effective [13], especially if the intervention entails app-embedded behavior-change techniques [18]. In addition, individually tailored feedback, based on the participant’s own characteristics, and advice is more likely to be effective compared with generic information about health benefits from PA [16, 19]. The Acupedo walking app will be used in the current study. The app is the number-one-ranked pedometer app at the app/google app store and comprises several behavior-change techniques, which is important when promoting PA [18]. In addition, participants will be encouraged to use an individually tailored home-based training app (three times/week) developed by the Swedish Military. This app comprises a pre-defined 12-weeks exercise program with progressively increasing exercise intensity. Both apps contain several behavior-change techniques: for example, self-monitoring and specific goal setting. Furthermore, participants will use food photography, via an app, a methodology shown to assess food intake in different environments with acceptable accuracy [20, 21]. Additionally, food photography holds promise as an intervention tool to change dietary decision-making and attitudes [22].

The intervention entails three face-to-face consultations, in groups of approximately 20 participants, held at the healthcare center. The first consultation, held at the start of the intervention (baseline), will focus on information on how to use the apps and improving motivation to increase walking and exercise, with additional discussions on goal setting and other techniques to support behavior change. The second PA consultation, held at week 6, will focus on semi-structured face-to-face interviews concentrating on facilitating and hindering factors associated with intervention adherence. Participants will review their progress towards achieving the goals set in the first meeting by discussing the information recorded on the walking app and the activity tracker. The final meeting at week 12 will focus on encouraging participants to maintain changes by reviewing goal attainment and discussing relapse prevention strategies to maintain healthy PA and other health habits. After 12 weeks, a purposive sample of participants are interviewed about their experiences of efficacy, implementation and outcomes of the app support and at 12 months with a focus on persistent changes of lifestyle.

### The supervised health program

#### Short description

The supervised health program is a 12-week standard care health program, delivered by health educators and personal trainers, including one weekly supervised exercise session (*n* = 55).

### Design

The supervised exercise program is based on the trans-theoretical and socio-cognitive models of behavior change [23]. The meetings with health educators and personal trainers within the intervention group will have a semi-structured format and a person-centered approach to ensure individualization to meet the needs of the participants with MD. To reduce the complexity of the meetings, a behavior-change model with four core behavior-change techniques (mobilizing social support for change, developing self-efficacy, goal setting and self-monitoring), that are known to be effective in supporting individuals to improve healthy activity and dietary behaviors [24] will be used. Although the focus will be on these four components, health educators and personal trainers will tailor the meetings by adding additional behavior-change techniques, such as identifying and overcoming barriers to change, and using a motivational interviewing approach where relevant. The health educators and personal trainers are experienced at adapting interventions to make them accessible to adults with MD.

Dietary advice given to the participants will follow the four-step Step-wise Weight-determined Accumulative change Plan (SWAP) model, which includes the following aspects: (1) limit sweets and snack intake to no more than 100 g/week, eaten during 1 day of the week, (2) substitute regular foods with low fat and low sugar alternatives, (3) gradually increase vegetable intake until vegetables cover half the plate at lunch and dinner and (4) reduce portion sizes by reducing equally from the carbohydrate and the fat content of the meal [25].

In addition to the dietary advice, participants will use a food photography app as described for the eHealth group. However, participants in the supervised exercise program group will also use the social media function imbedded in the app (a “locked” function made available only for this group), which enables sharing and rating of meals; thus, providing participants with social support, which has been shown to be beneficial to making behavioral changes [24].

The intervention will encompass both structured supervised and non-supervised PA including aerobic, strength, flexibility and balance training, and will be designed to be performed at fitness centers with personal trainers, one time per week with a total of 12 sessions during the intervention period. PA goals will be individualized based on each participant’s level of physical fitness (VO_2_max), and will further be modified in response to illness, injury or physical symptoms. Participants will be further encouraged to have an active lifestyle with two more weekly un-supervised exercise sessions. Participants will further be encouraged to avoid prolonged periods of uninterrupted sitting and aim at a minimum of 30 min daily walking. The weekly personal trainer supervised exercise sessions, lasting 60 min each, will target compound movements, involving large muscle groups. The exercise sessions during the first 4 weeks will aim at for a minimum of 10 min of PA at a moderate to vigorous intensity (> 60% VO_2_max). During the next 4 weeks, time in MVPA will progressively increase to 20 min per session and the last 4 weeks of the intervention will provide 30 min of MVPA per exercise session. Exercise sessions will be 60 min long throughout the intervention period. Thus, lower-intensity PA, such as balance and flexibility training, will be progressively replaced during the intervention period. The exercise sessions will be structured so that they can be generalized to the home environment; thus, possibly increasing post-intervention maintenance. Interviews with a purposive sample of participants will be performed at 12 weeks focusing on experiences of the efficacy, implementation and outcomes of the program and again at 12 months to explore persistent changes in lifestyle.

### Sample size calculation

Prior to this study, there were no objectively measured PA data from PA and exercise interventions for young adults with MD. A recent cross-sectional study with objectively measured MVPA showed an average of 11.6 min of MVPA per day (standard deviation 25.1) among individuals with MD [26]. A target between group difference of 10 min of daily MVPA, which has been shown to be a clinically relevant increase in activity, was used for the sample size calculation. For 80% power at the 5% significance level, 40 participants per group is required. To allow for a dropout rate of 20%, approximately 50 participants in each group will be required. Therefore, adopting a conservative approach, the final target sample size will be 55 participants in each treatment arm.

For the qualitative parts of this study, an estimated purposive sample is recruited. Participants are recruited until saturation of data is obtained.

### Study participants

A multi-point strategy will be applied to recruit a sufficient number of participants (in total 110 participants) with MD within a short timespan (approximately 1 month). Recruitment of participants will be performed by rehabilitation coordinators at county council rehabilitation centers, at primary care centers, rehabilitation centers and at private companies within the Stockholm area during early 2018. Participants will be given oral and written information about the trial at the rehabilitation centers and provide a written interest to participate. All participants will provide written informed consent before initializing the baseline assessments.

### Inclusion criteria

Currently, there are no agreed upon definitions of what constitutes a person with MD [26]. For the current study, participants are eligible if they are between 18 and 40 years of age with a MD, acquired within the past 3 years, and if they experience any mobility-related problems affecting their everyday life; for example, problems with dressing, performing household tasks, with transportation, personal hygiene tasks, or at work.

### Exclusion criteria

Exclusion criteria include being bound to a walker or wheelchair or having any medical condition not permitting moderate-intensity walking. Further exclusion criteria are not being able to speak/understand Swedish and not having access to a smartphone.

### Randomization

A block randomization procedure will be used at an individual level to ensure an equal distribution of participants between the two treatment groups. Participants will be randomized before the first baseline assessment at the healthcare center. The SAS Proc Plan (SAS Institute Inc., Cary, NC, USA) will be used for the randomization process. The block size will not be stated in the protocol so that the investigators are blind to the block size. Thus, limiting potential risk for selection bias.

### Baseline assessment

Participants will be invited to group consultation meetings, including baseline assessments, at a healthcare center. The group consultations will be held at three time points and include all participants recruited from the different recruitment sources.

After a short introduction, participants will perform a battery of physical tests and fill in web-based questionnaires lasting, altogether, approximately 2 h. Thereafter, participants will be randomized into the two treatment groups, followed by separate introductions for their treatment. In addition to the questionnaires described below, the participants will fill in questions on basic demographic information. The baseline measures and all follow-up measures will be made in the morning (fasted state) in order to best utilize standardize measures (e.g. blood samples).

### Primary outcome

The primary outcome, difference in time spent in MVPA per day at 12 weeks between the two groups, will be measured using the Actigraph GT3X+ tri-axial accelerometer (Actigraph, LCC, Pensacola, FL, USA), which has shown to accurately assess levels of PA under free-living conditions [27, 28]. Participants will be asked to wear the GT3X+ during all waking hours for seven consecutive days at baseline (week 0), midpoint (week 6), endpoint (week 12) and at maintenance follow-up (12 months). The minimum data requirement for inclusion in the analyses will be 10 h of data on at least 4 days, including one weekend day. Management and analyses of PA data will follow best practice and research recommendations [29]. In addition, we will analyze time spent in light and vigorous PA and sedentary time at all measure time points, as well as day-to-day data on steps/day from the participants’ smartphones (automatically stored in each smartphone “health kit” application).

### Secondary outcomes

Measures on secondary outcomes, aside from the semi-structured interviews on intervention adherence and enjoyment, will be taken at baseline (week 0), midpoint (week 6), endpoint (week 12) and at maintenance follow-up (12 months).

### Body composition measures

The physical tests include measures on body composition: height, weight, waist circumference, fat mass and fat-free mass via bioelectrical impedance [30]. Body mass and height will be measured with the participants wearing light clothes to the nearest 0.1 kg and 0.5 cm, respectively. An Omron model HBF-511B-E/HBF-511 T-E will be used for the bioelectrical impedance measures.

### Fitness tests

Fitness will be measured via a submaximal VO_2_max test performed on a stationary bike to assess aerobic fitness [31]. The participants will be instructed to cycle, with a pedal frequency of 60 rpm and a resistance of 0.5 kilopond (29 W), on a calibrated, mechanically braked, cycle ergometer (model 828E, Monark, Varberg, Sweden) during 4 min. Thereafter, the loading is progressively increased until the subject reaches a heart rate of 120 beats per minute. The mean pulse during the last minute is recorded. VO_2_max is the estimated by sex- and age-specific equations on differences in pulse rate between the higher and lower standard load.

### Blood sample measures

In addition, blood samples will be collected at weeks 0, and 12, as well as at 12 months. All blood samples will be procured by means of an intravenous cannula. At each sampling, the first 2 mL of the draw will be discarded as waste before a 5-mL sample is collected. This sample will then be dispensed in to an EDTA tube, a plasma-heparin tube and a serum tube. The two latter samples will be centrifuged at 1700 g for 20 min before initial storage in − 20 °C and later storage at − 80 °C pending subsequent analysis for genetic/epigenetic and metabolomics analysis. All blood samples will be sent to and stored at a Biobank at Karolinska Institutet.

Analyse of blood will include markers of inflammation and stress. Genetic single-nucleotide polymorphism and epigenetic methylation status genome-wide analysis will be performed using the Illumina-based platform [32] from blood samples taken at weeks 0, and 12. The genetic/epigenetic analyses will enable the current study to investigate the extent to which the intervention effects are driven by genetic/epigenetic factors, which, in twin studies, have shown to play a major role in responsiveness to PA within an intervention setting [33].

### Web-based questionnaire measures

Participants will answer a web-based survey measuring the following aspects:

- HRQoL by the SF-36 [34], a widely-used instrument which measures HRQoL with 36 questions divided in eight dimensions: physical functioning, role limitations due to physical health problems, bodily pain, general health, vitality, social functioning, role limitations due to emotional problems and mental health
- Musculoskeletal pain on a Visual Analogue Scale from zero (no pain) to 100 (worst imaginable pain) over the past week [35, 36]
- Perceived stress by the Perceived Stress Scale [37], a 10-item questionnaire (scored 0–4) based on how the respondent felt during the past month
- Symptoms of depression by the Beck Depression Inventory-II [38], one of the most widely used psychometric scales for measuring the severity of depression by a 21-item, multi-choice, self-report inventory
- Work ability via the Work Ability Index (WAI) scale [39], a questionnaire designed to quantify an individual’s capacity for work, comprising seven dimensions and including such questions as, “Assuming that your work ability at its best has a value of 10 points how many points would you give your current work ability?”
- Dietary intake by 24 h recall [40], a structured interview (administered by a trained interviewer) intended to capture detailed information about all foods and beverages consumed by the respondent in the past 24 h, from midnight to midnight the previous day
- Motivation towards PA and exercise will be measured by the Behavioral Regulation in Exercise Questionnaire [41], a 19-item questionnaire measuring the stages of the self-determination continuum with respect to motivation to exercise by a 5-point Likert scale (ranging from not true for me to very true for me)

### Qualitative interview measures

At the endpoint (week 12) and after 12 months, a purposive sample of participants from each arm will participate in individual semi-structured interviews or focus group discussions to qualitatively assess the efficacy, implementation and outcomes of the programs and their experiences of persistent lifestyle changes. All interviews are performed by trained health educators and digitally recorded. The interviews are transcribed to text verbatim and thereafter analyzed.

### Participant safety

Participant safety will be a main priority, and multiple strategies will be utilized to minimize adverse events associated with participation in the study. The supervised baseline testing session will ensure that participants are safe to participate in the planned intervention and assessments. Adverse events will be tracked closely, during meetings and research assessments, with special emphasis on events that could be associated with participation in the study.

## Statistical analyses

### Primary and secondary outcome measures

The primary study outcome, difference in levels of MVPA at 12 weeks between the two treatment groups, will be tested on an intention-to-treat approach, with all participants analyzed in the group to which they were randomized, using a two-tailed 0.05 significance level. We will further conduct per-protocol analysis to evaluate the effect on levels of MVPA at 12 weeks among those who followed the study protocols. Both intention-to-treat and per protocol analyses will be performed on differences in the main and secondary outcomes between the two groups at 12-week and 12-month follow-ups. Mixed-effects regression models will be used for analyses accounting for baseline levels of MVPA and possible clustering of the randomized groups. Similar regression models will be fitted for secondary outcomes. Data will be presented as interclass correlation coefficients, adjusted mean differences (95% confidence interval) and corresponding *p* values. For the primary outcome, within- and between-group changes will also be calculated from a repeated-measures mixed-effects model.

### Health economic evaluation

The cost-effectiveness analyses will be presented as additional cost per additional benefit, that is, additional SEK per health benefit gained, referred to as the incremental cost-effectiveness ratio (ICER). Health benefits include both primary (MVPA) and secondary outcomes and will also be evaluated as moving one individual from an inactive to an active category. Analyses will further account for the costs associated with the eHealth program and standard care supervised health program. Finally, data on HRQoL will be used to assess differences in quality-adjusted life years (QALYs) between the two intervention programs.

### Qualitative analysis

The qualitative data collection and analysis will have a grounded theory (GT) approach [42]. Transcribed data is analyzed according to the steps of GT focusing on the efficacy, implementation and outcomes of the programs and participants’ experiences of persistent lifestyle changes. Also, situational analysis (SA) may be applied; a method evolving from traditional GT. This analysis mainly follows the steps of GT, but SA allows the researcher to identify variations between the discourses and illustrate the socially and time-bound constructions that these discourses are used within. By using SA, three approaches (situational, social world/arena and positional maps) may be applied separately or together so as to communicate the variations in discourses.

## Discussion

The overall aim of the current intervention is to examine the efficacy of an eHealth program compared to a standard care supervised health program to support adults with MD to increase levels of PA and improve health-related behaviors.

### Significance

There is a lack of RCTs investigating effective ways to increase levels of PA in young adults with MD. Increased levels of PA among this physically inactive population have the potential to substantially improve health-related outcomes, possibly more so than in the general population [9]. The few existing studies suggest that motivation for PA and sports participation is high among individuals with MD and that barriers to PA engagement include accessibility to tailored PA, and most importantly, a lack of knowledge on how to engage in PA [6, 11, 12]. The current project aims to fill this gap in knowledge by examining effective and feasible methods to adopt a healthy and active lifestyle for young adults with MD by combining quantitative and qualitative methods. App-based programs are low-cost compared to physical trainers/health coaches. Also, app-based programs can reach most people, as most people in Sweden have smartphones with app-based abilities, while many people may lack money and/or access to physical trainers/health coaches. If a low-cost app-based program can effectively increase short- and long-term levels of PA, then large-scale implementation using apps within rehabilitation and medical centers, as well as workplace settings, is possible within a few years; thus, fundamentally changing general PA counseling approaches for individuals with MD. Further, if this intervention is found to be effective, there are great possibilities to implement the eHealth program to many other groups of inactive people within the population.

## Trial status

Requiting participants.

## Additional file

Additional file 1: Standard Protocol Items: Recommendations for Interventional Trials (SPIRIT) 2013 Checklist: recommended items to address in a clinical trial protocol and related documents*. (DOC 121 kb)

## Abbreviations

BMI: Body Mass Index
EPN: Regional Ethics Committee
GT: Grounded theory
HRQoL: Health-related quality of life
MD: Mobility disability
MVPA: Moderate to vigorous physical activity
PA: Physical activity

## References

[1] Health. SNIoP. Health on equal terms? Health and living conditions among people with disabilities. In: Health. SNIoP, editor. Socialstyrelsen: Swedish National Institute of Public Health; 2008.

[2] Calatayud J, Jakobsen MD, Sundstrup E, Casana J, Andersen LL (2015). Dose-response association between leisure time physical activity and work ability: Cross-sectional study among 3000 workers. Scand J Public Health.

[3] Arvidson E, Borjesson M, Ahlborg G, Lindegard A, Jonsdottir IH (2013). The level of leisure time physical activity is associated with work ability-a cross sectional and prospective study of health care workers. BMC Public Health.

[4] Sorensen LE, Pekkonen MM, Mannikko KH, Louhevaara VA, Smolander J, Alen MJ (2008). Associations between work ability, health-related quality of life, physical activity and fitness among middle-aged men. Appl Ergon.

[5] Warburton DE, Nicol CW, Bredin SS (2006). Health benefits of physical activity: the evidence. CMAJ.

[6] Saebu M, Sorensen M (2011). Factors associated with physical activity among young adults with a disability. Scand J Med Sci Sports.

[7] Carroll DD, Courtney-Long EA, Stevens AC, Sloan ML, Lullo C, Visser SN (2014). Vital signs: disability and physical activity— United States, 2009–2012. Mmwr-Morbid Mortal W.

[8] Dairo YM, Collett J, Dawes H, Oskrochi GR (2016). Physical activity levels in adults with intellectual disabilities: a systematic review. Prev Med Rep.

[9] van der Ploeg HP, van der Beek AJ, van der Woude LH, van Mechelen W (2004). Physical activity for people with a disability: a conceptual model. Sports Med.

[10] Pahor M, Guralnik JM, Ambrosius WT, Blair S, Bonds DE, Church TS (2014). Effect of structured physical activity on prevention of major mobility disability in older adults: the LIFE study randomized clinical trial. JAMA.

[11] Saebu M, Sorensen M, Halvari H (2013). Motivation for physical activity in young adults with physical disabilities during a rehabilitation stay: a longitudinal test of self-determination theory. J Appl Soc Psychol.

[12] Martin JJ (2006). Psychosocial aspects of youth disability sport. Adapt Phys Act Q.

[13] Schoeppe S, Alley S, Van Lippevelde W, Bray NA, Williams SL, Duncan MJ (2016). Efficacy of interventions that use apps to improve diet, physical activity and sedentary behaviour: a systematic review. Int J Behav Nutr Phys Act.

[14] Dennison L, Morrison L, Conway G, Yardley L (2013). Opportunities and challenges for smartphone applications in supporting health behavior change: qualitative study. J Med Internet Res.

[15] Turner L, Shamseer L, Altman DG, Weeks L, Peters J, Kober T (2012). Consolidated standards of reporting trials (CONSORT) and the completeness of reporting of randomised controlled trials (RCTs) published in medical journals. Cochrane Database Syst Rev.

[16] Foster C, Richards J, Thorogood M, Hillsdon M (2013). Remote and web 2.0 interventions for promoting physical activity. Cochrane Database Syst Rev.

[17] Samdal GB, Eide GE, Barth T, Williams G, Meland E (2017). Effective behaviour change techniques for physical activity and healthy eating in overweight and obese adults; systematic review and meta-regression analyses. Int J Behav Nutr Phys Act.

[18] Middelweerd A, Mollee JS, van der Wal CN, Brug J, Te Velde SJ (2014). Apps to promote physical activity among adults: a review and content analysis. Int J Behav Nutr Phys Act.

[19] Lustria ML, Noar SM, Cortese J, Van Stee SK, Glueckauf RL, Lee J (2013). A meta-analysis of web-delivered tailored health behavior change interventions. J Health Commun.

[20] Bouchoucha M, Akrout M, Bellali H, Bouchoucha R, Tarhouni F, Mansour AB (2016). Development and validation of a food photography manual, as a tool for estimation of food portion size in epidemiological dietary surveys in Tunisia. Libyan J Med.

[21] Martin CK, Nicklas T, Gunturk B, Correa JB, Allen HR, Champagne C (2014). Measuring food intake with digital photography. J Hum Nutr Diet.

[22] Zepeda L, Deal D (2008). Think before you eat: photographic food diaries as intervention tools to change dietary decision making and attitudes. Int J Consum Stud.

[23] Bandura A (2004). Health promotion by social cognitive means. Health Educ Behav.

[24] Teixeira PJ, Carraca EV, Marques MM, Rutter H, Oppert JM, De Bourdeaudhuij I (2015). Successful behavior change in obesity interventions in adults: a systematic review of self-regulation mediators. BMC Med.

[25] Huseinovic E, Winkvist A, Bertz F, Brekke HK (2014). Changes in food choice during a successful weight loss trial in overweight and obese postpartum women. Obesity (Silver Spring).

[26] Loprinzi PD, Sheffield J, Tyo BM, Fittipaldi-Wert J (2014). Accelerometer-determined physical activity, mobility disability, and health. Disabil Health J.

[27] Santos-Lozano A, Marin PJ, Torres-Luque G, Ruiz JR, Lucia A, Garatachea N (2012). Technical variability of the GT3X accelerometer. Med Eng Phys.

[28] Santos-Lozano A, Santin-Medeiros F, Cardon G, Torres-Luque G, Bailon R, Bergmeir C, et al. Actigraph GT3X: validation and determination of physical activity intensity cut points. Int J Sports Med. 2013. https://www.ncbi.nlm.nih.gov/pubmed/23700330. 10.1055/s-0033-1337945 23700330

[29] Migueles JH, Cadenas-Sanchez C, Ekelund U, Delisle Nystrom C, Mora-Gonzalez J, Lof M, et al. Accelerometer data collection and processing criteria to assess physical activity and other outcomes: a systematic review and practical considerations. Sports Med. 2017. https://www.ncbi.nlm.nih.gov/pubmed/28303543. 10.1007/s40279-017-0716-0 PMC6231536 28303543

[30] Kyle UG, Genton L, Karsegard L, Slosman DO, Pichard C (2001). Single prediction equation for bioelectrical impedance analysis in adults aged 20–94 years. Nutrition.

[31] Ekblom-Bak E, Bjorkman F, Hellenius ML, Ekblom B (2014). A new submaximal cycle ergometer test for prediction of VO2max. Scand J Med Sci Sports.

[32] Liu L, Li Y, Li S, Hu N, He Y, Pong R (2012). Comparison of next-generation sequencing systems. J Biomed Biotechnol.

[33] Zadro JR, Shirley D, Andrade TB, Scurrah KJ, Bauman A, Ferreira PH (2017). The beneficial effects of physical activity: is it down to your genes? A systematic review and meta-analysis of twin and family studies. Sports Med Open.

[34] Ware JE, Sherbourne CD (1992). The MOS 36-item short-form health survey (SF-36). I. Conceptual framework and item selection. Med Care.

[35] Farrar JT (2000). What is clinically meaningful: outcome measures in pain clinical trials. Clin J Pain.

[36] Farrar JT, Portenoy RK, Berlin JA, Kinman JL, Strom BL (2000). Defining the clinically important difference in pain outcome measures. Pain.

[37] Cohen S, Kamarck T, Mermelstein R (1983). A global measure of perceived stress. J Health Soc Behav.

[38] Wang YP, Gorenstein C (2013). Psychometric properties of the Beck Depression Inventory-II: a comprehensive review. Rev Bras Psiquiatr.

[39] de Zwart BC, Frings-Dresen MH, van Duivenbooden JC (2002). Test-retest reliability of the Work Ability Index questionnaire. Occup Med (Lond).

[40] Beer-Borst S, Amado R (1995). Validation of a self-administered 24-hour recall questionnaire used in a large-scale dietary survey. Z Ernahrungswiss.

[41] Markland D, Tobin V (2004). A modification to the behavioural regulation in exercise questionnaire to include an assessment of amotivation. J Sport Exercise Psy.

[42] Strauss A, Corbin J (2015). Basics of qualitative research: techniques and procedures for developing grounded theory.

